# Design and Assessment of Flexible Capacitive Electrodes for Reusable ECG Monitoring: Effects of Sweat and Adapted Front-End Configuration

**DOI:** 10.3390/s25185856

**Published:** 2025-09-19

**Authors:** Ivo Iliev, Georgi T. Nikolov, Nikolay Tomchev, Bozhidar I. Stefanov, Boriana Tzaneva

**Affiliations:** 1 Department of Electronics, Faculty of Electronic Engineering and Technology, Technical University of Sofia, Kliment Ohridski Blvd., 8, 1000 Sofia, Bulgaria; gnikolov@tu-sofia.bg (G.T.N.); nntomchev@gmail.com (N.T.); 2 Department of Chemistry, Faculty of Electronic Engineering and Technology, Technical University of Sofia, Kliment Ohridski Blvd., 8, 1000 Sofia, Bulgaria; borianatz@tu-sofia.bg

**Keywords:** flexible electrode, capacitive ECG, non-contact biopotential sensing, impedance spectroscopy, Kapton dielectric, moisture effects

## Abstract

This work presents the development and characterization of a flexible capacitive electrode for non-contact ECG acquisition, fabricated using a simple and cost-effective method from readily available materials. The electrode consists of a multilayer structure with a copper conductor laminated by a polyimide (Kapton^®^) dielectric layer on a polyurethane support. The impedance and capacitance of the electrode were evaluated under varying textile moisture levels with artificial sweat, as well as after exposure to common disinfectants including ethyl alcohol and iodine tincture. Electrochemical impedance spectroscopy (EIS) and broadband impedance measurements (10^−1^–10^5^ Hz) confirmed stable capacitive behavior, moderate sensitivity to moisture, and chemical stability of the Kapton–copper interface under conditions simulating repeated use. A custom front-end readout circuit was implemented to demonstrate through-textile ECG signal acquisition. Simulator tests reproduced characteristic waveform patterns, and preliminary volunteer recordings confirmed the feasibility of through-textile acquisition. These results highlight the promise of the electrode as a low-cost platform for future wearable biosignal monitoring technical research.

## 1. Introduction

Healthcare systems in developed countries, including Bulgaria, are increasingly challenged by the unprecedented aging of the population. According to the World Health Organization (WHO), by 2030, one in six people globally will be aged 60 years or older, and this number is projected to double to 2.1 billion by 2050. In Europe and North America, one in four individuals will fall into this age group [1]. As a result, social and healthcare expenditures are rising sharply, driven by the need to maintain health and quality of life in the aging population.

To address this issue, new technologies are being developed to shift healthcare delivery beyond traditional clinical settings—toward home- and community-based care. One promising solution is remote monitoring (telemetry) of vital signs for patients who do not require immediate hospitalization but still need regular observation to manage chronic conditions. This approach is especially valuable in rural or underserved areas where access to specialized care is limited [2].

Systematic reviews [3] have highlighted the growing adoption of wearable technologies for remote health monitoring, particularly in elderly populations. Commonly tracked parameters include heart rate, body temperature, motion, respiratory rate, blood glucose, oxygen saturation, and blood pressure. Comparative studies of commercial and prototype devices have evaluated their suitability for such applications. A related review [4] emphasized ongoing challenges in material selection, sensor design, and power supply—particularly the need for flexible, multifunctional, and biocompatible materials.

Given that cardiovascular diseases account for 32% of all global deaths [5,6], remote monitoring technologies are increasingly focused on cardiac function. Early screening and monitoring are critical, and wearable or non-contact electrocardiographic (ECG) systems are gaining traction [7,8]. When integrated with information and communication technologies (ICT) and artificial intelligence (AI), these systems can support preliminary assessment and long-term observation.

Despite these advancements, remote ECG monitoring cannot yet replace standard diagnostic tools such as clinical electrocardiographs and long-term Holter monitors. Successful use of out-of-hospital ECG systems requires that users be in sufficiently good physical and cognitive condition to operate the device, maintain regular data acquisition, and, most importantly, correctly position the electrodes. Therefore, ongoing research aims to simplify ECG self-recording, with much of the effort focused on electrode development.

Traditional Ag/AgCl gel electrodes, widely used in clinical settings, provide low-impedance skin contact but suffer from gel drying over time, leading to signal degradation. Gel and adhesive components may also cause skin irritation or allergic reactions [9,10,11]. To address these issues, researchers have proposed dry electrodes made from various conductive materials. These eliminate the need for gel and generally improve user comfort [12,13,14,15,16]. Some designs, such as the micro-spiked electrodes by Griss et al. [17] or the 3D-printed conical structures by Salvo et al. [18], aim to reduce impedance on hairy skin, though they remain susceptible to motion artifacts. Other innovations include the thin Ti/Au composite electrodes on flexible substrates by Baek et al. [19] and the hexagonal labyrinth pattern proposed by Elango et al. [20], which was found to offer superior skin contact and long-term performance. Still, even dry electrodes require direct contact between conductive material and skin.

An alternative approach utilizes galvanically isolated (non-contact) electrodes that operate primarily through capacitive coupling with the body. In this context, the capacitive ECG (cECG) architecture consists of a conductive electrode separated from the body by a dielectric layer, forming a capacitor in which the body acts as the second electrode plate. This configuration enables biopotential signal acquisition without direct electrical contact, reducing skin irritation and improving user comfort. A key advantage of the cECG approach is its ability to capture ECG signals through clothing, allowing for seamless integration of electrodes into textile-based systems and expanding the potential for wearable, unobtrusive health monitoring applications. Nevertheless, cECG has inherent limitations: it cannot measure absolute signal amplitudes reliably, very low-frequency components (below 1 Hz) are strongly attenuated, variability between measurements can be extreme, calibration across sessions is problematic, and motion artifacts are significantly more severe than in standard ECG. These limitations restrict the immediate clinical applicability of cECG and require careful consideration when interpreting experimental results.

The main technical challenge in cECG is the transmission of low-amplitude (1–5 mV), low-frequency (0.05–150 Hz) signals through a capacitance typically in the 10–100 pF range [21]. Depending on the specific purpose of the diagnostic examination, a low frequency of 0.05 Hz is determined as the limit value, and for rhythm interpretation, the accepted frequency is 0.67 Hz [22]. Such conditions demand extremely high input impedance from the signal acquisition front end. Conventional buffer stages based on operational amplifiers often include a high-value resistor (>100 GΩ) to stabilize input potential, but these resistors are costly, prone to noise, and difficult to source [23,24]. Alternative methods include using T-shaped resistor networks or bootstrap amplifier configurations [23,25], typically employing amplifiers with >1 TΩ input impedance, <2 pF input capacitance, high common-mode rejection, and minimal bias currents.

Badarov et al. [26] proposed a simplified bootstrap design using JFET op-amps that mitigates parasitic capacitance and signal drift but requires wider power supply ranges—limiting its use in compact wearable systems. While these circuit solutions make cECG feasible, practical challenges remain, particularly motion artifacts and equipment-induced noise. Power-line interference (PLI) can be minimized through shielding, guarding, and right-leg drive circuits [23,27]. Some researchers also propose adaptive filtering using electrode-tissue impedance as a reference signal [28].

Nonetheless, long-term stability remains a concern, primarily due to variations in electrode capacitance that determine the input impedance of the amplifier. These variations complicate calibration and, consequently, the accurate measurement of ECG signal amplitudes (see justification below). As noted, the small values of electrode capacitance directly affect the accuracy of recording low-frequency ECG components (below 1 Hz) and, in particular, the faithful reproduction of diagnostically important elements such as the ST segment. Motion artifacts (MAs) are another major issue in cECG recording. Various approaches for MA suppression have been reported, including the following: (1) the use of digital filtering methods implemented with DSP [29] and (2) adaptive noise filtering, where the Electrode–Tissue Impedance (ETI) is used as a reference signal [30]. In a comprehensive review on MAs in capacitive ECG monitoring systems, Khalili et al. [28] noted that most of the proposed solutions require substantial computational resources and/or additional circuitry (sensors and measurement channels, etc.), which in turn increase power consumption and often limit their applicability in wearable devices. According to the authors, a promising direction for future research lies in approaches that combine digitally controlled analog designs, which may prevent amplifier saturation and signal loss.

In addition to motion-related disturbances, a number of less obvious but equally critical factors, such as ambient humidity, electrode flexibility, and moisture content, especially in textile-based dielectrics, can strongly influence signal quality [31]. Moisture, in particular, tends to reduce impedance and enhance signal acquisition [32]. Several studies recommend maintaining optimal humidity conditions [9,31,33,34]. Cui et al. [35] showed that high humidity combined with controlled pressure improves signal quality and impedance consistency. It is reasonable to assume that sweat could have a similar effect; however, the influence of its chemical composition on signal integrity and electrode longevity during continuous or repeated use requires further study.

This work presents the evaluation of a flexible capacitive ECG electrode, comprising a copper conductor insulated by a polyimide (Kapton^®^, produced by DuPont, Wilmington, DE, USA) dielectric layer on a polyurethane support, with a focus on its stability and performance under varying moisture exposure. The feasibility of through-textile ECG signal acquisition is examined here as a technical demonstration by testing the electrode’s impedance characteristics and electrochemical behavior in conditions simulating real-world use, including exposure to artificial sweat and contact with common disinfectants such as ethanol and iodine tincture. Particular attention is given to the effects of repeated wetting and cleaning, assessing the electrode’s durability and functional reliability. In parallel, a dedicated hardware solution is implemented to provide real-time digital adjustment of amplifier gain asymmetry, enabling improved common-mode interference suppression and enhancing signal quality during non-contact ECG measurements through clothing.

## 2. Materials and Methods

### 2.1. Flexible Capacitive Electrode Implementation and Testing Protocol

**Figure 1:**
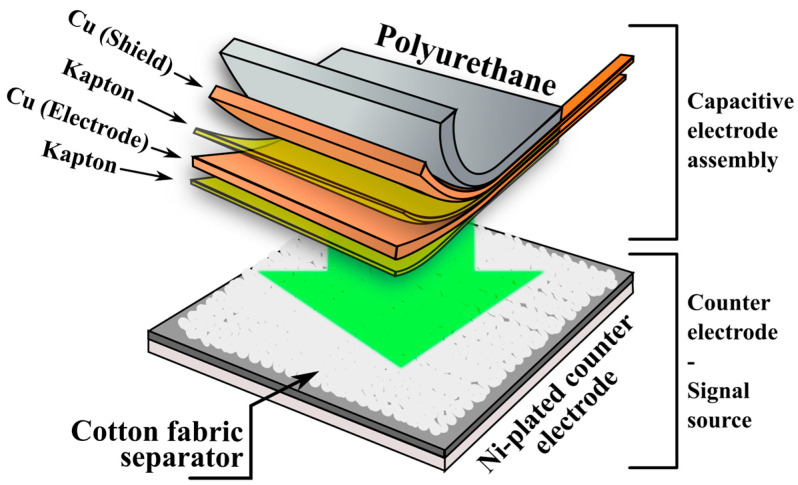
Schematic representation of the capacitive multilayer electrode structure and the test configuration, including the cotton textile separator and the Ni-plated counter-electrode, used as a signal source.

The capacitive electrode evaluated in this study consists of a flexible multilayer structure, schematically illustrated in Figure 1.

The capacitive electrode was formed from copper (Cu) foil (50 × 50 × 0.05 mm), laminated onto a Kapton insulating sheet, which also acts as the dielectric barrier in the capacitive interface. The copper layer serves as the conductive plate of the capacitor, while the human body acts as the opposing plate during ECG signal acquisition. A secondary Cu/Kapton bilayer is included for electrical shielding. The complete multilayer structure was laminated onto a polyurethane-based flexible substrate (50 × 50 × 0.7 mm) to provide mechanical support and ensure even pressure distribution during testing. Based on the bill of materials, the raw material cost of one such electrode (50 × 50 mm) is approximately EUR 0.37, with unit costs of ~EUR 23.5/m^2^ for copper tape, ~EUR 9.9/m^2^ for Kapton tape, and ~EUR 78.2/m^2^ for polyurethane sheets. For comparison, single-use Ag/AgCl electrodes typically cost in the range of EUR 0.50–1.02 per unit, depending on the fabrication method, while carbon-based printed electrodes are reported at EUR 0.56 per unit, and laser-induced graphene (LIG) electrodes can reach as low as EUR 0.27 per unit [36]. This demonstrates the cost-efficiency of the proposed reusable electrode design.

A textile separator was used in all test setups to simulate a clothing interface, mimicking conditions for through-garment ECG acquisition. The separator was made of knitted cotton fabric (jersey-type, 50 × 50 × 0.7 mm), chosen for its hygroscopic properties. The counter-electrode was fabricated using a nickel-plated FR-4 substrate to ensure chemical resistance in the artificial sweat environment. During testing, the capacitive electrode was pressed into contact with the textile layer and positioned against the counter-electrode under a constant pressure of approximately 425 g-force (1.67 kPa). This configuration was used across all electrochemical and frequency-dependent impedance measurements, as well as in ECG signal acquisition tests using a simulator.

To assess changes in the electrical properties of the electrode under moisture exposure, a model solution of artificial sweat was used. The formulation contained 20 g L^−1^ NaCl, 17.5 g L^−1^ NH_4_Cl, 5 g L^−1^ acetic acid, and 15 g L^−1^ D,L-lactic acid, following ISO 3160-2:2015 recommendations for testing the corrosion resistance of metals in contact with skin [37]. Tests using the “dry textile” separator were conducted under ambient atmospheric conditions, at a relative humidity of 55 ± 5% and room temperature (25 ± 3 °C). Under these conditions, the naturally adsorbed moisture in the textile (moisture regain of cotton of 6.61%) is neglected and is therefore referred to in this work as “dry textile”. Artificial sweat was applied to the textile separator using a fine mist sprayer in volumes ranging from 0.2 mL to 0.8 mL, evenly distributed over the entire 25 cm^2^ surface area. Accordingly, the volume of sweat per unit area varied from 8 μL cm^−2^ to 32 μL cm^−2^, corresponding to a moisture content of approximately 26% to 58% when normalized to the weight of the textile separator (573 mg). The upper value of 32 μL cm^−2^ corresponds to the maximum liquid retention capacity of the textile separator (before dripping) and to the intense sweating rate in the chest area of an adult individual after physical exertion [38].

To simulate cyclic reuse conditions and evaluate disinfectant compatibility, additional tests were conducted on electrodes subjected to standard cleaning agents. These included rubbing alcohol (aqueous ethyl alcohol, 98% *v*/*v*) and tincture of iodine (5 wt. % I_2_, 2 wt. % KI in 50 *v*/*v* aqueous ethyl alcohol), which were applied to the Kapton surface for a duration of 30 min, followed by rinsing with distilled water and thorough drying. The high concentrations of the disinfectants along with the application of a long-duration disinfection treatment significantly exceeded typical medical practice in order to simulate long-term use. The effects of these treatments on the electrode’s performance and surface properties were further evaluated through impedance and wetting angle measurements.

### 2.2. Investigation of Impedance Characteristics of the Capacitive Electrode

To characterize the frequency-dependent impedance response of the electrode and assess its stability under moisture exposure, two complementary methods were employed. The first method involved a conventional indirect measurement approach, in which the voltage drop across the device under test (DUT) was measured directly, and the current was calculated by monitoring the voltage drop across a precision reference resistor connected in series with the DUT. The data was complemented by electrochemical impedance spectroscopy (EIS) using a potentiostat–galvanostat system.

**Figure 2:**
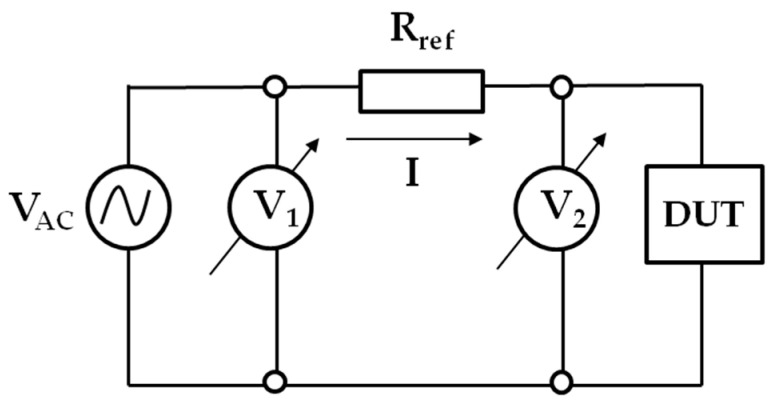
Simplified circuit for measuring the impedance of a DUT by evaluating two voltage drops.

The low-frequency impedance behavior of the capacitive electrode, a classical indirect measurement method, was employed. In this configuration, the voltage drop across the capacitive electrode (DUT). The current is then calculated indirectly by monitoring the voltage drop across a precision reference resistor $R_{ref}$ connected in series with the DUT. A simplified schematic of the method is shown in Figure 2.

An AC voltage source of relatively low frequency, labeled $V_{AC}$, drives the system. The complex impedance of the DUT can be calculated from the voltage drops $V_{1}$ and $V_{2}$, and the known reference resistor $R_{ref}$, using the following relation:

(1) $$Z_{DUT}=\frac{V_{2}}{V_{1}-V_{2}}R_{ref}$$

To determine the magnitude and phase angle of the impedance, the system is analyzed using phasor representation in polar coordinates [39,40]. The magnitude of the impedance and the phase shift φ between the voltage signals are determined via the law of cosines and trigonometric relationships as follows:

(2) $$\left|Z_{DUT}\right|=\frac{V_{2}}{\sqrt{V_{1}^{2}+V_{2}^{2}-2V_{1}^{2}V_{2}^{2}\cos\theta }}R_{ref}$$

(3) $$\varphi =arctg\frac{-V_{2}\sin\theta }{V_{1}-V_{2}\cos\theta }$$

The overall impedance phase angle α is obtained by subtracting φ from the measured phase difference θ, as follows:

(4) $$\alpha =\theta -\varphi =\theta -arctg\frac{-V_{2}\sin\theta }{V_{1}-V_{2}\cos\theta }$$

To extract the equivalent series resistance $R_{ESR}$ and capacitance C from the complex impedance, the impedance is converted back to rectangular form as follows:

(5) $$Z=R_{ESR}-\frac{j}{2\pi fC}$$

(6) $$Z=\left|Z\right|\cos\alpha +j\left|Z\right|\sin\alpha ,$$

where f is the frequency of the AC signal. From this, the real and imaginary components yield the following:

(7) $$R_{ESR}=\left|Z\right|\cos\alpha$$

(8) $$C=\frac{-1}{2\pi f\left|Z\right|\sin\alpha }$$

**Figure 3:**
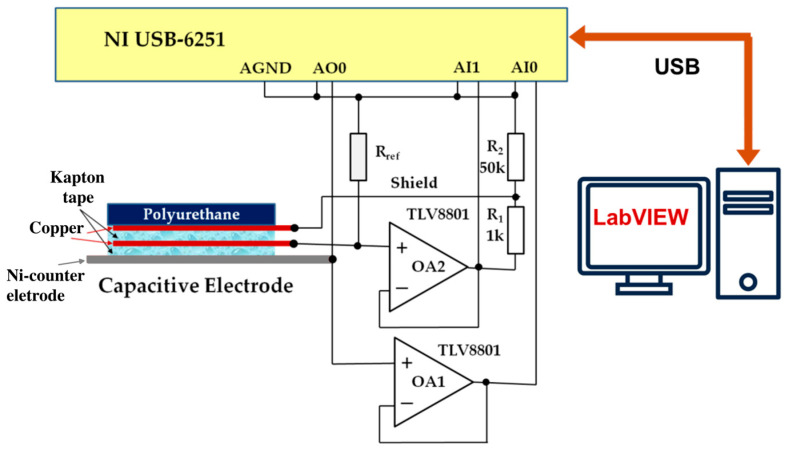
Block diagram of the experimental setup of a virtual system for impedance measurement.

The experimental setup for this analysis is illustrated in Figure 3.

The system is based on a National Instruments USB-6251 data acquisition (DAQ) system. To minimize the influence of the DAQ input impedance, operational amplifiers OA1 and OA2 (TLV8801, Texas Instruments) are used in the front end. Amplifier OA2 additionally provides shield-driving voltage to the capacitive electrode via a voltage divider ($R_{1}$ and $R_{2}$). The reference resistor $R_{ref}$ is switchable depending on the measurement frequency: 1 GΩ is used for the 0.1–10 Hz range, and 100 MΩ for the 10–100 Hz range.

To probe the extended frequency range (10^−1^–10^5^ Hz), additional measurements with electrochemical impedance spectroscopy were performed without external DC polarization, using a sinusoidal excitation voltage with an amplitude of 50 mV. Measurements were conducted with a PalmSens4 potentiostat–galvanostat, and equivalent circuit modeling was pe1rformed using PSTrace 5.9 software.

The tests were carried out on two identical capacitive electrodes, with 5 to 8 measurements for each condition. Electrodes were evaluated immediately after fabrication (“fresh”), after extended use involving repeated impedance measurements and ECG signal acquisition (“used”), and following contact with textiles soaked in two common disinfectant solutions. The disinfectant treatments included 30-min contact with ethyl alcohol and 30-min contact with tincture of iodine. In all cases, the textile separator and counter-electrode configuration were consistent with that described in Section 2.1.

These measurements aimed to assess the chemical and electrochemical stability of the Kapton-based electrode structure during prolonged exposure to sweat and repeated cleaning—conditions representative of wearable sensor reuse. Equivalent circuit models were used to extract impedance parameters such as interfacial resistance, capacitance of the dielectric barrier, and constant phase element behavior, which are discussed in detail in Section 3.1. and summarized in Supplementary Table S1.

### 2.3. ECG Signal Acquisition Using a Simulator and Practical Demonstration

To evaluate the functional performance of the capacitive electrode and illustrate feasibility and signal detectability in capturing biopotential signals, ECG tests were conducted using both a commercial patient simulator and healthy volunteers. Simulator-based ECG recordings were performed through the controlled cotton textile layer to demonstrate through-clothing measurement capability. For the volunteer tests, the electrodes were placed over the participants own garments as a demonstration of signal acquisition through everyday clothing.

**Figure 4:**
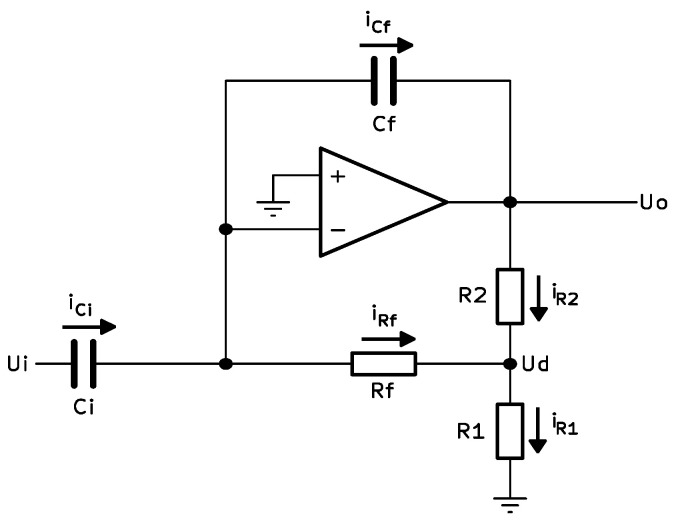
Schematic of the input amplifier stage featuring a T-shaped feedback network. The gain is determined by the electrode-to-feedback capacitance ratio.

The custom front-end readout circuit developed for this purpose utilizes charge-sensitive amplifiers at the input stage, incorporating T-network feedback structures as shown in Figure 4.

The gain for this amplifier stage is determined through the ratio of the capacitance of the electrode ($C_{i}$), and the feedback capacitance ($C_{f}$). The feedback T-network consists of resistors $R_{f}$, $R_{1}$, and $R_{2}$ forming an equivalent resistance $R_{eq}$ across the feedback path.

Starting with Kirchoff current law and Ohm’s law:

(9) $$i_{R_{2}}=i_{R_{1}}-i_{R_{f}}$$

(10) $$i_{R_{f}}=-\frac{U_{d}}{R_{f}}$$

(11) $$i_{R_{1}}=-\frac{U_{d}}{R_{1}}$$

(12) $$U_{d}=-i_{R_{f}}.R_{f}$$

(13) $$U_{o}=R_{2}.i_{R_{2}}+U_{d}$$

Substituting (10) and (11) into (9):

(14) $$i_{R_{2}}=\frac{U_{d}(R_{f}+R_{1})}{R_{1}R_{f}}$$

Combining (12) and (14):

(15) $$i_{R2}=-\frac{i_{Rf}\left(R_{f}+R_{1}\right)}{R_{1}}$$

Substituting into (13):

(16) $$U_{o}=-i_{Rf}\left(\frac{R_{2}(R_{f}+R_{1})}{R_{1}}+R_{f}\right)$$

Rearranging:

(17) $$i_{R_{f}}=-\frac{U_{o}}{R_{f}\left(\frac{R_{2}}{R_{1}}+1\right)+R_{2}}=-\frac{U_{o}}{R_{eq}}$$

Now, considering the currents through the capacitors:

(18) $$i_{C_{i}}=C_{i}\frac{dU_{i}\left(t\right)}{dt},$$

(19) $$i_{C_{f}}={-C}_{f}\frac{dU_{C_{f}}\left(t\right)}{dt}$$

(20) $$i_{C_{i}}=i_{C_{f}}+i_{R_{f}}$$

Substituting (17)–(19) into (20), and taking Laplace transforms:

(21) $$C_{i}sU_{i}=-C_{f}.s.U_{o}-\frac{U_{o}}{R_{eq}}$$

Thus, the gain $U_{o}$/$U_{i}$ can be expressed as:

(22) $$\frac{U_{o}}{U_{i}}=-\frac{s.C_{i}.R_{eq}}{s.C_{f}.R_{eq}-1}$$

Finally, assuming $sC_{f}R_{eq}\gg 1$, the gain simplifies to:

(23) $$\frac{U_{o}}{U_{i}}=\frac{C_{i}}{C_{f}}$$

This relationship shows that the amplifier gain depends on the ratio of the input and feedback capacitances, with minimal influence from the resistive elements under typical operating frequencies. In practice, however, this dependence of the gain on electrode capacitance represents a limiting factor for the calibration of cECG recorders and, consequently, for the accurate measurement of the amplitude components of the electrocardiogram.

For ECG signal generation, a PS2240 ECG Patient Simulator, dedicated to testing and calibration of ECG units, was used to output standardized ECG waveforms, including normal sinus rhythm and various arrhythmias, with adjustable amplitude and frequency. The simulator output was routed through the textile-interfaced capacitive electrode setup shown in Figure 1.

In addition to the simulator tests, a practical signal acquisition was carried out using the same electrode and readout system on two healthy volunteers (a 23-year-old male and a 22-year-old female). The participants were laboratory collaborators, and their electrocardiograms were recorded with personal informed consent and approval from the university ethics committee.

**Figure 5:**
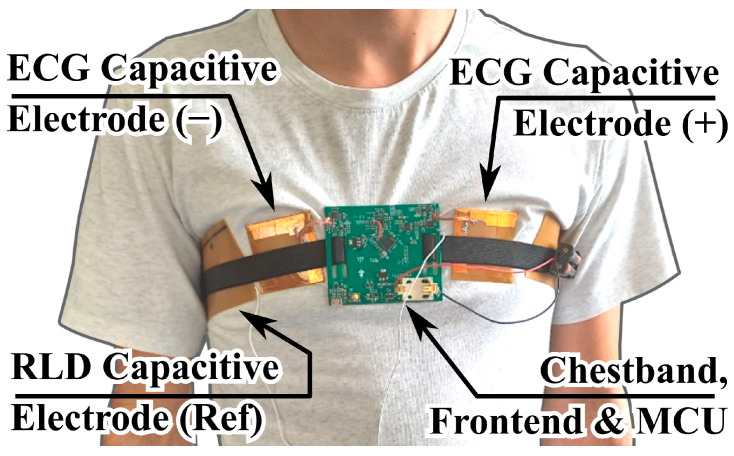
Setup for a practical demonstration of through-textile cECG acquisition employed in measurements on volunteers.

The setup used for these measurements is shown in Figure 5.

As illustrated, two capacitive electrodes were employed, pressed against the chest of the test subject via an elastic chest band, which also served as a support for the analog front end (described in later sections) and the readout electronics with microcontroller unit (MCU). A separate flexible electrode provided the reference right-leg drive (RLD) signal for common-mode interference suppression. The recorded ECG signals were acquired with the electrodes positioned over a cotton T-shirt, rather than through direct skin contact.

The volunteer measurements in this study represent a preliminary demonstration of applicability, aimed at validating the functionality of the proposed capacitive electrode structure and front-end configuration under realistic usage conditions and allowing for a side-by-side evaluation between simulator-generated and real biopotential signals.

## 3. Results and Discussion

### 3.1. Impedance Behavior of the Capacitive Electrode from 0.1 Hz to 100 Hz

**Figure 6:**
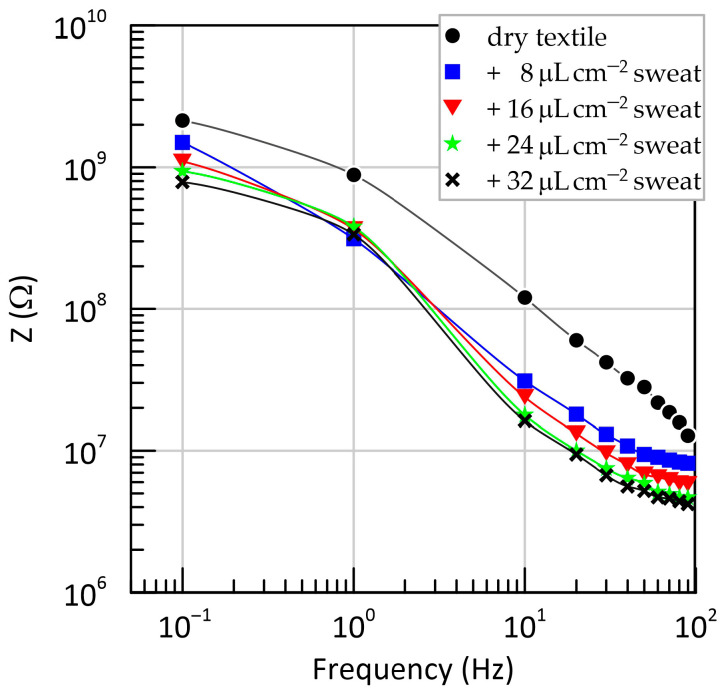
Impedance magnitude of the capacitive electrode as a function of frequency for different levels of artificial sweat added to the textile separator.

To characterize the frequency-dependent behavior of the flexible capacitive electrode, impedance was measured across a wide frequency range (0.1–100 Hz) with the data presented in Figure 6.

The plot demonstrates a characteristic capacitive response, with impedance decreasing monotonically with increasing frequency. At low frequencies (below 10 Hz), the dry configuration exhibits impedance values above 1 GΩ, highlighting the high resistivity of the dielectric interface in the absence of moisture. Upon adding even a small amount of artificial sweat (8 μL cm^−2^), the impedance drops by nearly an order of magnitude in the sub-10 Hz region, confirming that ionic conductivity plays a significant role in coupling efficiency.

With further moisture addition (16–32 μL cm^−2^), the impedance decreases slightly but progressively less, and the curves begin to overlap, especially above 10 Hz. This plateau indicates that the textile separator reaches moisture saturation at ~16 μL cm^−2^, beyond which its conductive properties remain relatively unchanged.

Overall, these measurements verify the strong capacitive behavior of the electrode across all tested conditions and confirm that even small levels of perspiration can significantly reduce interface impedance. This reduction in impedance is a consequence of increased capacitance, which in turn facilitates the registration of low-frequency ECG components. At the same time, this effect may also introduce variability and potential distortion at very low frequencies.

### 3.2. Electrochemical Impedance Spectroscopy for Evaluating the Chemical Stability of the Electrode

The aim of the electrochemical tests was to evaluate whether the flexible electrode can be reliably used as a capacitive ECG electrode for long-term and repeated application. For consistent performance in such applications, the electrode must maintain high interfacial resistance that remains stable during prolonged exposure to chemically aggressive fluids, such as human sweat or disinfectant solutions. The high salt content and mildly acidic pH of sweat may compromise the barrier properties of the polyimide film, potentially allowing electrolyte penetration to the copper conductor and initiating undesirable oxidation processes. These could introduce parasitic noise in the acquired ECG signals. Additional concerns stem from typical disinfectants, which often contain organic solvents like ethanol or strong oxidizers such as iodine compounds and hydrogen peroxide, all of which could affect the integrity of the polyimide–copper interface.

**Figure 7:**
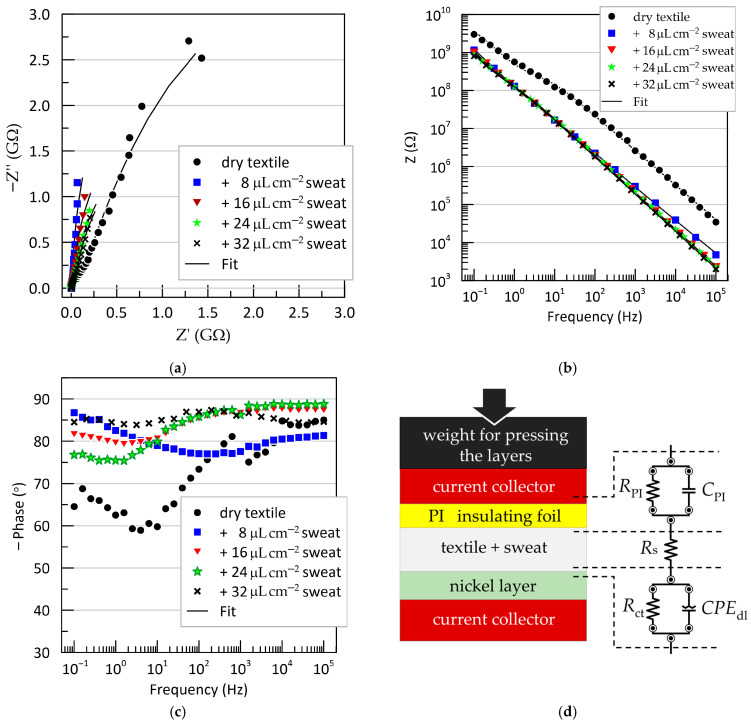
EIS results for the tested electrode using a textile separator with different quantities of artificial sweat: (**a**) Nyquist plots; (**b**) Bode impedance plots; (**c**) Bode phase angle plots; (**d**) equivalent circuit used to fit the EIS data. $R_{PI}$—resistance of the polyimide foil; $C_{PI}$—capacitance of the polyimide foil; $R_{s}$—resistance of the sweat-soaked textile separator; $R_{ct}$—charge transfer resistance; $CPE_{dl}$—constant phase element representing the electric double layer at the Ni/textile interface.

Electrochemical impedance spectroscopy (EIS) results obtained from a “fresh” electrode, tested under varying degrees of wetting of the textile separator with artificial sweat, are shown in Figure 7. The data are presented as Nyquist and Bode plots for the sandwich configuration: test electrode/textile (dry or wetted with artificial sweat)/Ni-plated copper counter electrode. The Nyquist plots (Figure 7a) and the frequency dependence of the impedance magnitude (Figure 7b) both exhibit typical capacitive behavior, with experimental points forming nearly linear trajectories. However, the phase angle–frequency relationships (Figure 7c) reveal the presence of two time constants, indicating that a more complex equivalent circuit is needed beyond a single ideal capacitor.

The remaining EIS measurements, performed after various treatments of the Kapton film, showed no significant deviations compared to the untreated “fresh” electrode (see Supplementary Figure S1).

In addition to the qualitative characterization of electrode behavior, quantitative interpretation of the EIS results requires fitting to an equivalent circuit. Various circuit models for capacitive ECG electrodes have been proposed in the literature [28,32,41,42,43]. However, their direct application is not suitable in our case, as these models describe the electrode-body interface, whereas our objective is to assess the chemical resistance of materials in contact with sweat. The simplest equivalent circuit of an electrochemical interface is the parallel resistor-capacitor (RC) model in series with the electrolyte resistance [41,44]. We adopted this model, with the final equivalent circuit shown in Figure 7d. It provided an excellent fit to the experimental data and consists of the resistance of the sweat-soaked textile separator ($R_{s}$), positioned between two RC blocks. The first RC branch ($R_{PI}C_{PI}$) corresponds to the Kapton@Cu/textile interface, while the second branch ($R_{ct}CPE_{dl}$) represents the textile/Ni interface. Instead of a pure capacitance, the electric double-layer capacitance at the metal-electrolyte interface is modeled using a constant phase element ($CPE_{dl}$), described by the following:

(24) $$Z_{CPE}=\frac{1}{Y_{0}{\left(i\omega \right)}^{n}},$$

as opposed to the ideal capacitor:

(25) $$Z=\frac{1}{i\omega C_{dl}}$$

here, $Y_{0}$ is a pseudo-capacitance parameter (with units Ω^−1^s^n^ = Fs^n−1^), and n is a dimensionless factor ranging from 0 to 1 that accounts for deviations from ideal capacitive behavior. The origin of the CPE element is typically attributed to surface phenomena on the metal electrode in contact with the electrolyte—such as surface roughness, passivation, and specific adsorption—which lead to non-uniform distributions of resistance, capacitance, and current density across the interface [44].

**Figure 8:**
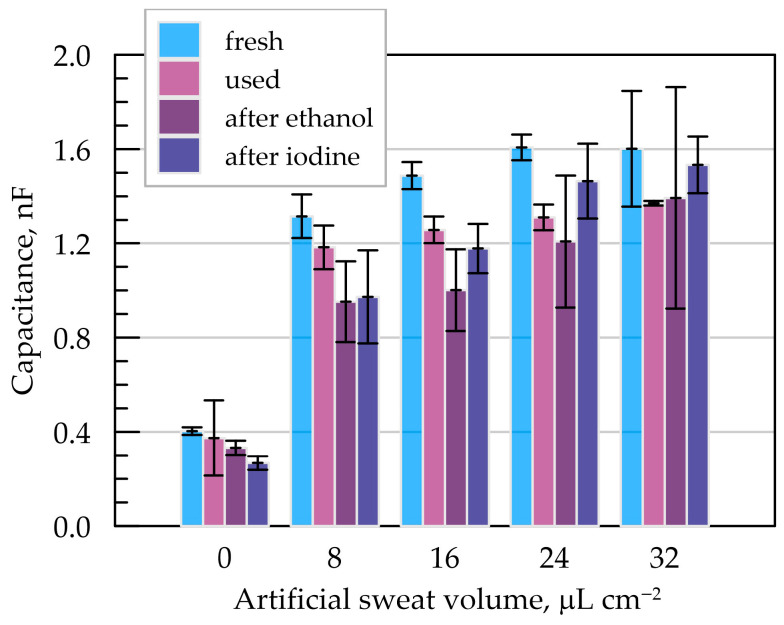
Influence of textile wetting with artificial sweat on the $C_{PI}$ values determined via EIS.

The extracted values for all equivalent circuit elements under different conditions—varied artificial sweat levels and surface treatments—are listed in Supplementary Table S1. The change in the interfacial capacitance $C_{PI}$ (polyimide interface) is graphically presented in Figure 8.

As expected, the largest difference in $C_{PI}$ occurs between the dry textile condition and all other sweat-wetted configurations. The dry system exhibited the lowest capacitance values, ranging from 0.2 to 0.6 nF. Even minimal wetting of the textile with an ionic medium (artificial sweat) caused a sharp increase in $C_{PI}$ above 1 nF. Interestingly, the capacitance increases only moderately with further increases in sweat volume—from 8 μL cm^−2^ (semi-dry) to 32 μL cm^−2^ (fully soaked), with total variation around 0.3 nF. Beyond 16 μL cm^−2^, $C_{PI}$ remains effectively constant, suggesting saturation behavior.

EIS tests were also performed on the same electrodes after prolonged use, including repeated sweat exposure and mechanical bending. In these “used” samples, the $C_{PI}$ values were slightly lower than those of the fresh electrodes, with the deviation becoming more noticeable at higher moisture levels. For dry textiles, the decrease was around 30 pF, while for sweat-soaked samples, $C_{PI}$ was reduced by up to 0.3 nF.

Surface treatment with iodine did not significantly affect $C_{PI}$, with variations remaining within the margin of experimental error. Ethanol treatment resulted in a slight decrease in polyimide capacitance and increased uncertainty in the measurements. To better assess the extent of interaction between the electrode surface and the different fluids, contact angle measurements were also performed after 30 min of exposure to artificial sweat, ethanol, and iodine solutions. The results, shown in Supplementary Figure S2, indicate a marginal improvement in wettability following contact with iodine and sweat and a slight deterioration after ethanol exposure.

It should be emphasized that these surface treatments represent more aggressive and prolonged exposure than would be expected under typical disinfection conditions in wearable sensor applications. In conclusion, the Kapton-based capacitive electrodes demonstrate adequate mechanical robustness and chemical resistance to artificial sweat, ethanol, and iodine tincture, indicating their promise for long-term material durability in wearable sensor platforms.

### 3.3. ECG Signal Acquisition with the Capacitive Electrode System

**Figure 9:**
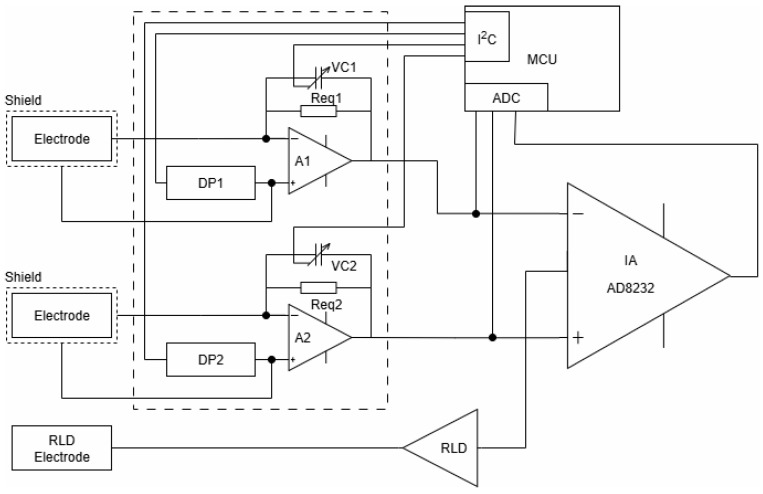
Block diagram of the developed front-end measurement system for capacitive ECG signal acquisition. The design includes charge-sensitive amplifiers, common-mode suppression, and baseline correction elements.

The applicability of the developed capacitive electrodes for electrocardiographic (ECG) signal acquisition was explored through laboratory demonstrations using standardized signals from an ECG simulator and through experimental recordings from volunteers under real contact conditions. Figure 9 shows the block diagram of the front-end measurement system based on the widely used module AD8232 (IA), specially developed for ECG and other biopotential measurement applications. It is designed to extract, amplify, and filter small biopotential signals in the presence of noisy conditions, such as those created by motion or remote electrode placement. The integrated right-leg drive (RLD) amplifier further suppresses the power line interference.

To minimize the influence of variable parameters of the capacitive electrodes, charge-sensitive amplifiers (A1 and A2) are included at the input stage. These amplifiers incorporate T-network feedback structures, as illustrated in Figure 4. As was mentioned above, the gain of these amplifiers is determined from the ratio of the input (electrode) and feedback capacitances. In practice, the gain in the proposed schematic solution is digitally adjustable via NCD2400MTR variable capacitors (VC1 and VC2) in the feedback loops of the amplifiers. This adjustment is managed by a PIC32MX150F128D microcontroller (MCU), which monitors the amplitude of the common-mode signal at the output of the IA and corrects (via I2C interface) the amplification of both amplifiers in a manner that reduces the imbalance in the input contours relating to common-mode signals.

This correction compensates for small variations in capacitance between the electrodes, especially when the textile interface is relatively dry. The gain adjustment remained within ±5% for properly mounted electrodes under realistic conditions, ensuring both signal fidelity and amplitude accuracy in controlled test environments.

**Figure 10:**
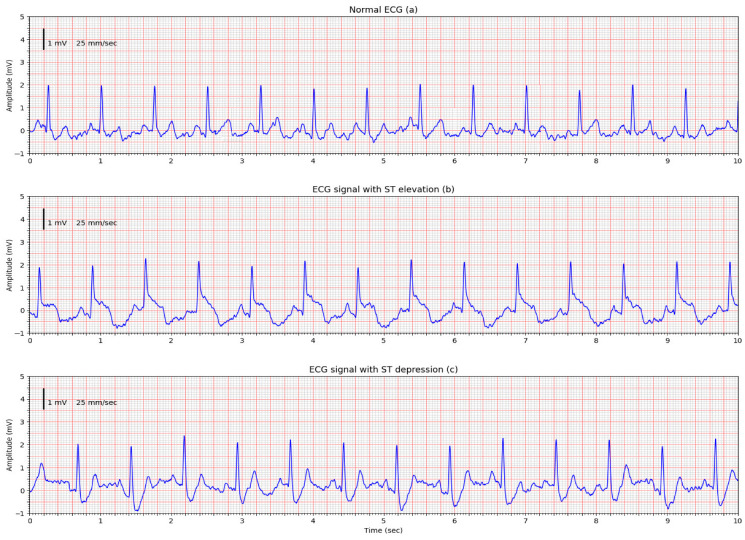
ECG signals recorded from the PS2240 simulator using the capacitive electrode system: (**a**) Normal ECG waveform; (**b**) ECG with ST-segment elevation (+300 μV); (**c**) ECG with ST-segment depression (−300 μV). The system reproduces characteristic waveform features of simulator outputs.

Figure 10 presents the recorded waveforms from a PS2240 ECG simulator (BC Marketplace). The top trace corresponds to a standard ECG waveform with a 2 mV peak amplitude. The middle and bottom traces show signals with ST-segment elevation and depression, respectively. We chose to test the reproducibility of these cardiac pathologies because they are indicative of the low-frequency range of the amplifier, dependent on the capacitance of the electrodes and the input impedance of the amplifiers.

As can be seen, the simulator-induced waveform variations were distinguishable. The degree of elevation or depression shown in the plots cannot be regarded as absolutely reliable, since calibration of the measurement system sensitivity (mV/cm) is not possible. What can be stated, however, is that the signals exhibit characteristic changes in the ST-segment region, which should be interpreted only as indicative and requiring precise confirmation through conventional ECG diagnostics. A comparison of these measurements was also performed with those obtained using a traditional in-hospital patient monitor (AnyView P12). The corresponding control records are provided in the Supplementary Information (Figure S3) and demonstrate a good overlap in signal shape under simulator conditions with the waveforms acquired by the capacitive sensor setup. A more pronounced difference is observed in the baseline recovery of signals with ST-segment elevation, which can be attributed to the lower cutoff frequency (0.5 Hz) of the developed recording system.

The system also incorporates a digital baseline correction mechanism. This is achieved using MCU-controlled (via I2C interface) digital potentiometers CAT5171 (DP1 and DP2) that adjust the DC offset at the inputs of the buffer amplifiers based on real-time feedback. This approach optimizes the amplifier’s dynamic range, allowing operation even under intensive baseline wander.

**Figure 11:**
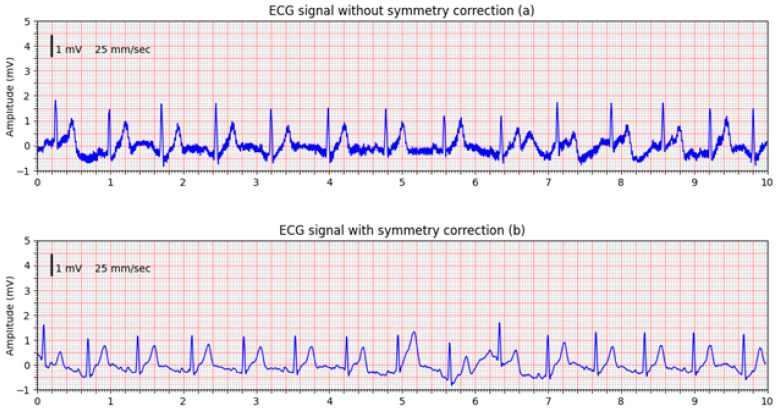
ECG signals recorded from volunteers: (**a**) ECG signal without symmetry correction; (**b**) ECG signal recorded with active feedback control.

In addition to simulator-based testing, the system’s ability to record real ECG signals was demonstrated using measurements from two healthy volunteers. Figure 11 shows a comparison between ECG signals acquired without symmetry correction (Figure 11a) and with active symmetry correction enabled (Figure 11b). The ECGs were acquired without changing the electrode positions, and the comparison confirms the effectiveness of the designed front-end circuitry in suppressing common-mode interference and improving signal quality.

**Figure 12:**
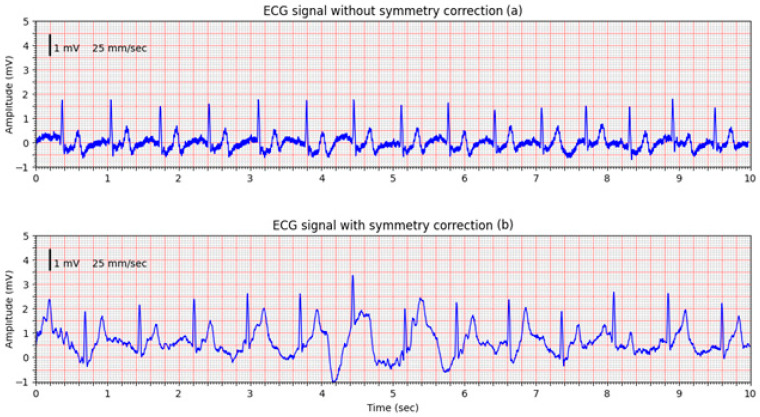
ECG signals recorded from a healthy volunteer: (**a**) ECG signal without symmetry correction; (**b**) ECG signal recorded with active feedback control, showing transient motion artifact. The system compensates for baseline drift in proof-of-concept tests.

This is confirmed in a second volunteer trial, where the improvement of the non-corrected signal (Figure 12a) is obvious, however, an intentional movement of the volunteer introduced a pronounced motion artifact visible in the middle of the signal (Figure 12b). The system’s DC offset adjustment mechanism in the input amplifiers responded promptly, successfully compensating for the baseline shift. Despite the motion-induced disturbance, the system maintained a usable signal, suitable for demonstrating technical robustness.

**Figure 13:**
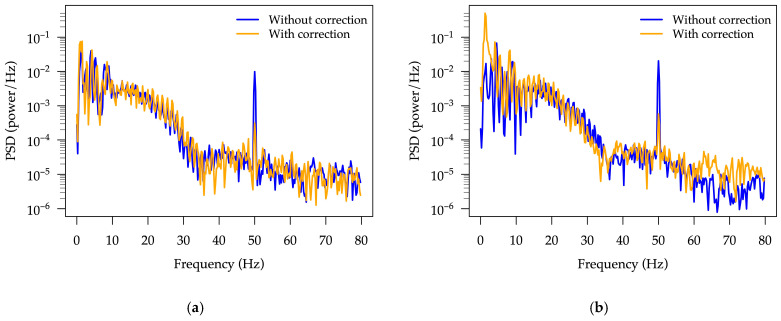
PSD plots for the ECG data presented in: (**a**) Figure 11; and (**b**) Figure 12. The clear suppression of the 50 Hz common-mode interference is notable when symmetry correction is active.

A power spectral density (PSD) analysis was carried out on the data presented in Figure 11 and Figure 12 using Hann windowing, 800-sample segments with 50% overlap. The resulting PSD spectra in the 0–80 Hz frequency range, obtained with and without symmetry correction, are shown in Figure 13. As can be seen, a pronounced suppression of the 50 Hz common-mode interference is achieved when symmetry correction is enabled, demonstrating the ability of the adapted front-end circuitry to reduce interference.

**Figure 14:**
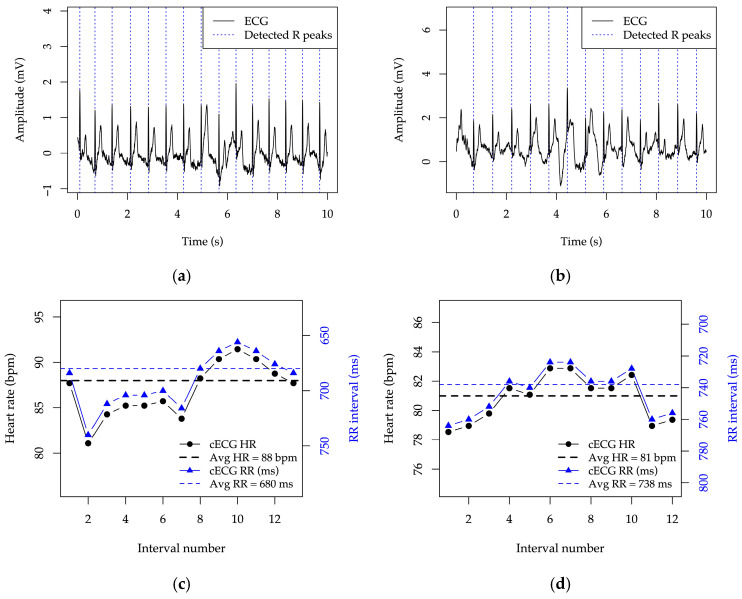
Demonstration of R-peak detection from the ECG signals and rhythm analysis based on the estimated R-R interval, based on the data shown in: (**a**,**c**) Figure 11; and (**b**,**d**) Figure 12.

The system reacts so as to keep the signal within the measurable range and to allow reliable recognition of R peaks, enabling subsequent rhythm analysis of the signal, as illustrated in Figure 14.

## 4. Conclusions

This study demonstrates the viability of a flexible, cost-effective capacitive electrode for non-contact ECG signal acquisition, fabricated from readily available materials using a simple multilayer Kapton-insulated Cu foil structure on a flexible polyurethane structural support. Its performance was evaluated through a cotton textile interface, highlighting the electrode’s ability to function reliably even when signals are acquired through clothing.

Electrochemical impedance spectroscopy (EIS) and broadband impedance measurements (10^−1^–10^5^ Hz) confirmed that the electrode retains its capacitive behavior and chemical stability after repeated exposure to artificial sweat and common disinfectants such as ethanol and iodine tincture. A significant increase in interfacial capacitance was observed with moisture saturating beyond 16 μL cm^−2^, which correlates with the sweat-retention capacity of the textile separator rather than the absolute sweat volume.

The custom-designed readout system, incorporating digitally adjustable gain and baseline correction, enabled robust ECG signal acquisition from both a simulator and human subjects. Simulator-based measurements reproduced characteristic waveform patterns. However, due to the absence of a guaranteed sensitivity criterion for the measurement system, low-frequency components such as ST-segment deviations can only be regarded as indicative of changes in cardiac activity, which would require confirmation through conventional clinical equipment. Volunteer recordings illustrated proof-of-concept feasibility under everyday clothing conditions.

Overall, the results indicate the potential of this low-cost, reusable electrode design as a technical platform for future wearable ECG research. While promising for unobtrusive signal monitoring through clothing, the inherent limitations of cECG, e.g., amplitude inaccuracy, low-frequency attenuation, variability between sessions, and strong susceptibility to motion artifacts, preclude clinical interpretation at this stage. A serious challenge remains the calibration of cECG recording systems: given the variable nature of the electrode–body coupling capacitance, calibration would need to be performed immediately prior to ECG acquisition. Further studies with larger cohorts, simultaneous reference ECG acquisition, and standardized signal quality assessment will be essential before any diagnostic use can be considered

## Data Availability

The data are not publicly available due to data privacy regulations. The data presented in this study are available on request from the corresponding author.

## Supplementary Materials

The following supporting information can be downloaded at: https://www.mdpi.com/article/10.3390/s25185856/s1, Table S1: Equivalent electrical circuit parameters from EIS fitting for all electrodes; Figure S1. EIS results for tested electrode after varied treatments; Figure S2. Variation in contact angles for water on the fresh Kapton surface, and after exposure to synthetic sweat, ethanol and iodine treatment; Figure S3. Control records of the PS2240 simulator generated ECG patterns, measured with an AnyView P12 patient monitor, corresponding to the data presented in Figure 10.
