# Supplementary material for: Design and Assessment of Flexible Capacitive Electrodes for Reusable ECG Monitoring: Effects of Sweat and Adapted Front-End Configuration
Source: Sensors (Basel). 2025 Sep 19;25(18):5856. doi: 10.3390/s25185856 (PMC12473565; doi:10.3390/s25185856)
Supplement: Supplementary file 1 [file sensors-25-05856-s001.zip › sensors-3841284-supplementary.pdf]

Supplementary Materials

Design and Assessment of Flexible Capacitive Electrodes for Reusable ECG Monitoring: Effects of Sweat and Adapted Front-End Configuration

Ivo Iliev [1,*], Georgi T. Nikolov [1], Nikolay Tomchev [1], Bozhidar I. Stefanov [2,*] and Boriana Tzaneva [2]

[1] Department of Electronics, Faculty of Electronic Engineering and Technology, Technical University of Sofia, Kliment Ohridski Blvd., 8, 1000 Sofia, Bulgaria; izi@tu-sofia.bg (I. I.); gnikolov@tu-sofia.bg (G. N.); nntomchev@gmail.com (N. T.).
[2] Department of Chemistry, Faculty of Electronic Engineering and Technology, Technical University of Sofia, Kliment Ohridski Blvd., 8, 1000 Sofia, Bulgaria; borianatz@tu-sofia.bg (B. Tz.); b.stefanov@tu-sofia.bg (B. I. S).
* Correspondence: izi@tu-sofia.bg

The equivalent electrical circuit for fitting of EIS results is denoted by the circuit description code ($R_{PI}C_{PI}$)R_S($R_{ct}CPE_{dl}$), where R_{PI} and C_{PI} correspond to the resistor and capacitance of the Kapton@Cu/textile interface, R_S is the resistance of the sweat-soaked textile separator and ($R_{ct}CPE_{dl}$) represents the textile/Ni interface. In the case of a dry textile separator, its natural humidity is neglected and R_S is removed from the circuit to give ($R_{PI}C_{PI}$)($R_{Ni}CPE_{Ni+separator}$). The values of the equivalent circuit parameters are presented in Table S1.

Table S1. Equivalent electrical circuit parameters from EIS fitting for all electrodes.

Artificial sweat	Electrode surface	R_{ct}, MΩ	CPE_{dl}, nF s^{n-1}	n	R_s, Ω	R_{PI}, MΩ	C_{PI}, nF	χ^2
**0 μL cm^{-2} (dry textile)**	fresh	259.5	0.259	0.88	–	8962	0.404	0.0542
	used	96.4	0.699	0.80	–	10,552	0.374	0.0220
	treated with ethanol	135.24	0.549	0.83	–	29,133	0.333	0.0239
	treated with iodine	220.6	0.174	0.91	–	7012	0.268	0.0173
**8 μL cm^{-2}**	fresh	14.3	4.518	0.82	26	13,600	1.315	0.0033
	used	33.1	5.918	0.86	176.9	12,050	1.183	0.0022
	treated with ethanol	674.1	3.864	0.90	75.6	19,353	0.952	0.0012
	treated with iodine	119.6	3.548	0.86	126.1	18,405	0.973	0.0004
**16 μL cm^{-2}**	fresh	26.0	3.107	0.93	22	5972	1.488	0.0013
	used	28.7	4.784	0.87	57.9	15,355	1.258	0.0006
	treated with ethanol	84.9	3.377	0.93	65.4	12,446	1.001	0.0006
	treated with iodine	43.8	2.850	0.93	26.0	11,140	1.178	0.0011
**24 μL cm^{-2}**	fresh	48.2	2.242	0.95	10.0	3841	1.608	0.0020
	used	14.0	7.077	0.90	41.1	22,800	1.311	0.0003
	treated with ethanol	90.5	1.986	0.95	10.7	4928	1.208	0.0012
	treated with iodine	51.1	5.148	0.90	27.2	13,430	1.465	0.0004
**32 μL cm^{-2}**	fresh	81.4	2.201	0.96	10.0	6909	1.602	0.0019
	used	17.73	7.198	0.89	53.4	12,200	1.371	0.0006
	treated with ethanol	102.0	2.345	0.95	16.5	4013	1.393	0.0020
	treated with iodine	52.2	7.532	0.89	96.5	7073	1.534	0.0006

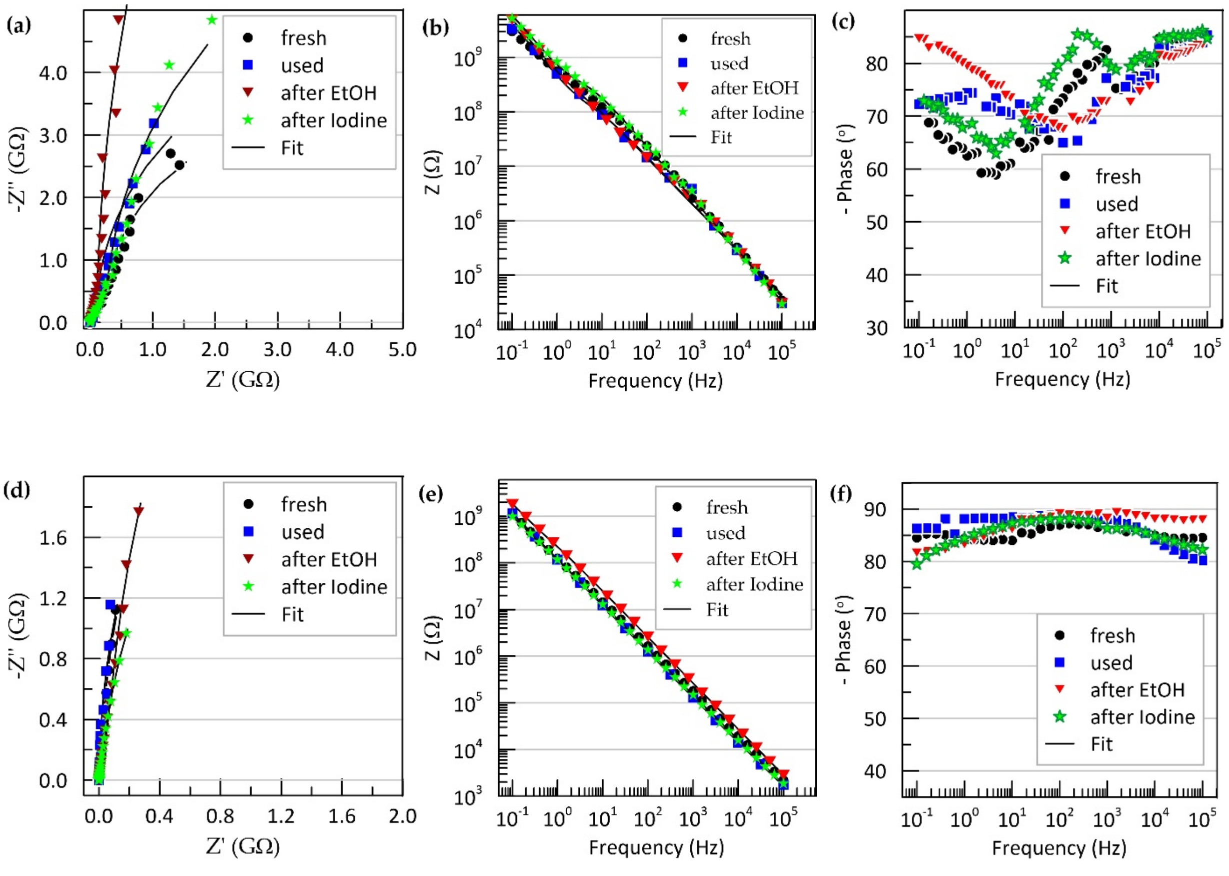

Figure S1. EIS results for tested electrode using dray textile separator (a, b, and c) and with 32 μL/cm^2 sweat (d, e, and f) after different treatments. Nyquist plots **(a, d);** Bode impedance plots **(b, e);** Bode phase angle plots **(c, f)**.

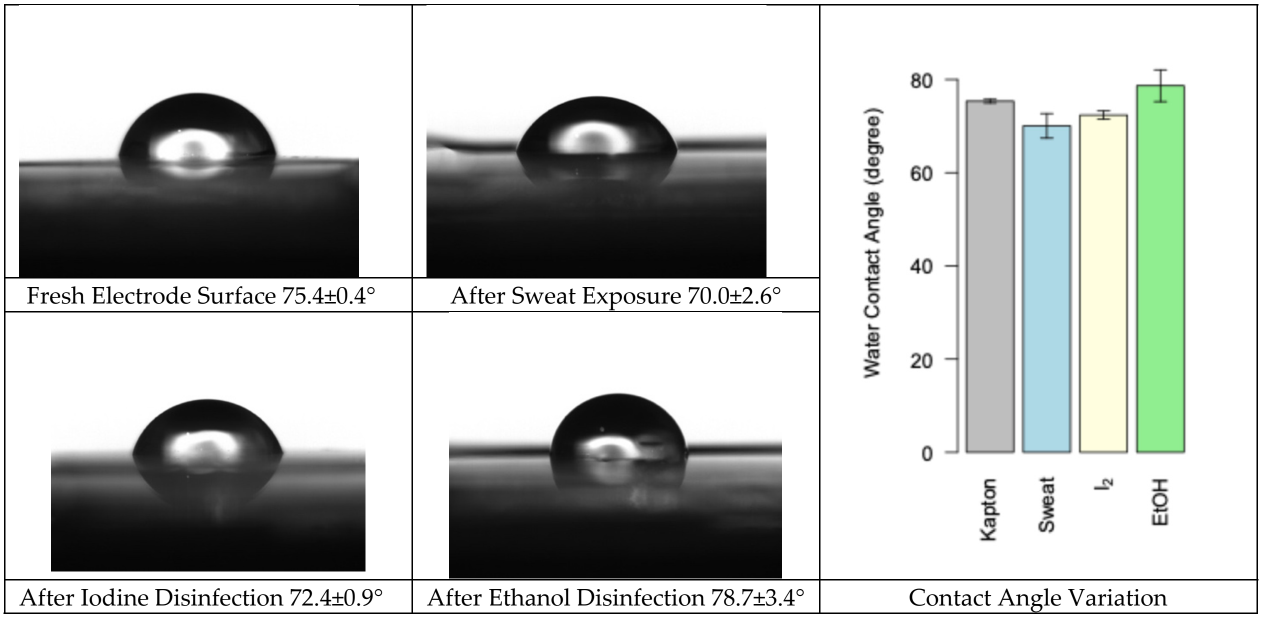

Figure S2. Variation in contact angles for water on the fresh Kapton surface, and after exposure to synthetic sweat, ethanol and iodine treatment.

(a)

(b)

(c)

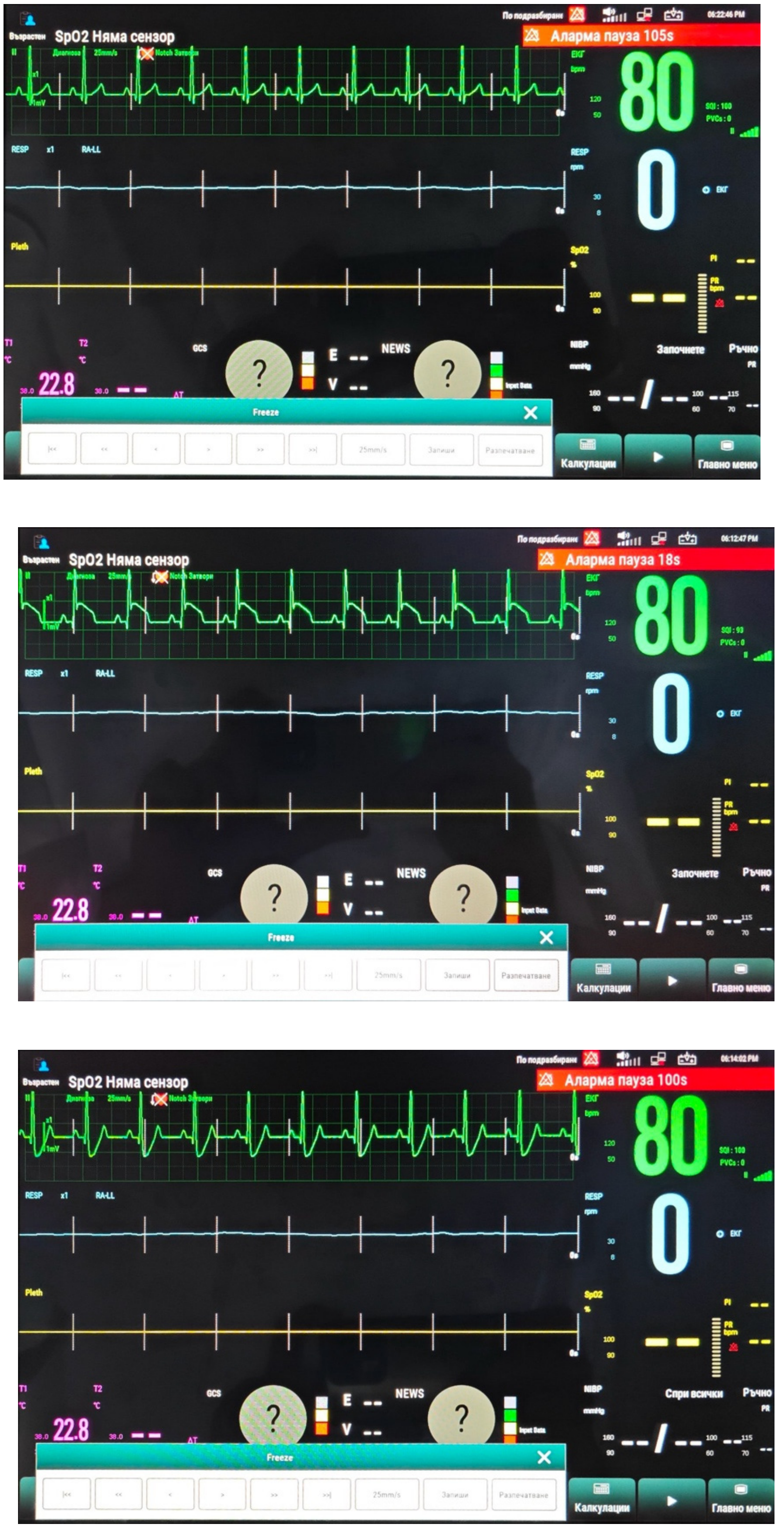

Figure S3. Control records of the PS2240 simulator-generated ECG patterns, measured with an AnyView P12 patient monitor, corresponding to the data presented in Figure 10: **(a)** normal ECG; **(b)** ECG with ST-segment elevation (+300 μV); **(c)** ECG with ST-segment depression (−300 μV).
